# Supplementary material for: Determination of the spin-lifetime anisotropy in graphene using oblique spin precession
Source: Nat Commun. 2016 May 9;7:11444. doi: 10.1038/ncomms11444 (PMC4865811; doi:10.1038/ncomms11444)
Supplement: Supplementary Information — Supplementary Figures 1-7, Supplementary Notes 1-6 and Supplementary References [file ncomms11444-s1.pdf]

I. SUPPLEMENTARY FIGURES

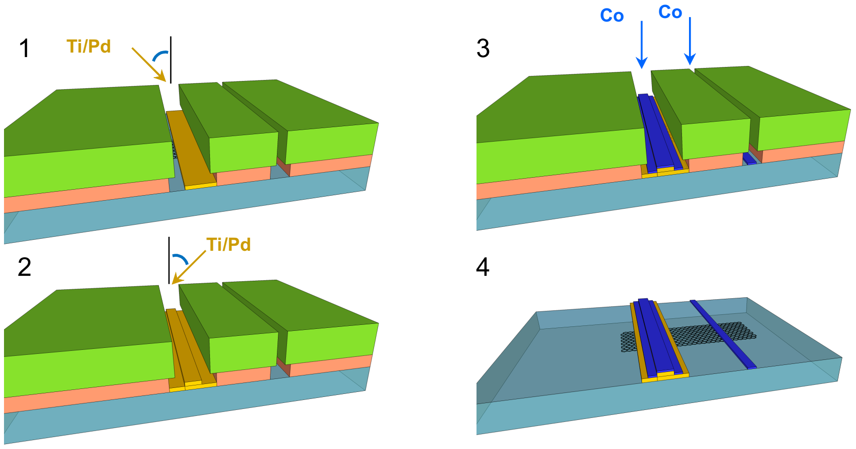

Supplementary Figure 1. Device fabrication. Partial view of the design of the shadow mask made with e-beam lithography using a PMMA/MMA bilayer on a SiO_2/Si substrate. Only the left half of the device is shown, the right half is a mirror image of the left half. The metal deposition sequence is shown by the steps 1 to 3. The inner Ti/Pd electrodes are deposited first at ±45°, then the outer Co electrodes are deposited under normal incidence. The final layout after lift-off is shown in step 4.

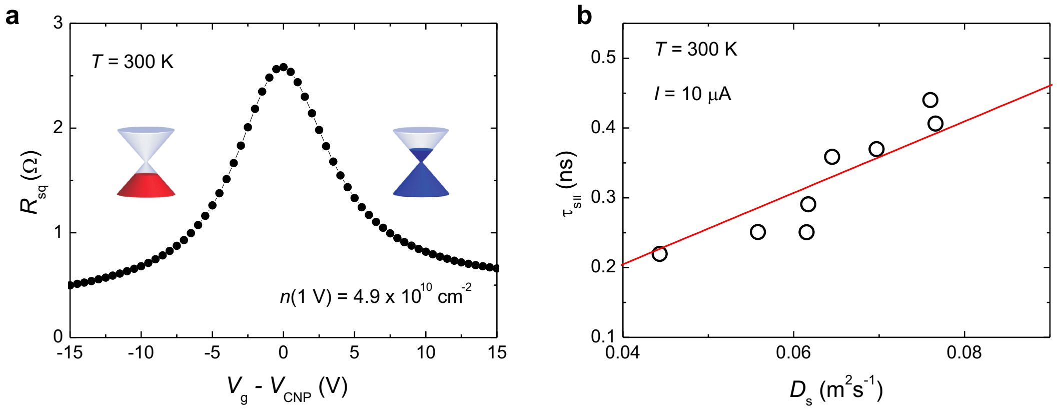

Supplementary Figure 2. Transport characteristics of the graphene device. **a**, Square resistance R_{sq} of the graphene channel as a function of gate voltage V_{g} at room temperature. The charge neutrality point is positioned at $V_{\mathrm{CNP}} = -22.5$ V. **b**, Extracted $\tau_{\mathrm{s}\|}$ vs D_{s} from spin relaxation parameters of the main text, the line is a linear fit.

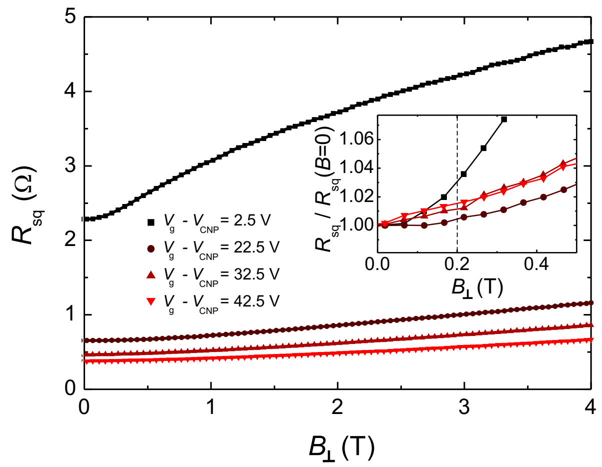

Supplementary Figure 3. Graphene magnetoresistance. Graphene square resistance $R_{\rm sq}$ versus magnetic field $B_\perp$ up to 4 T and up to 0.5 T (inset). In the latter, $R_{\rm sq}$ is normalized. The dashed vertical line in the inset shows the maximum field range in which the oblique spin precession measurements are performed.

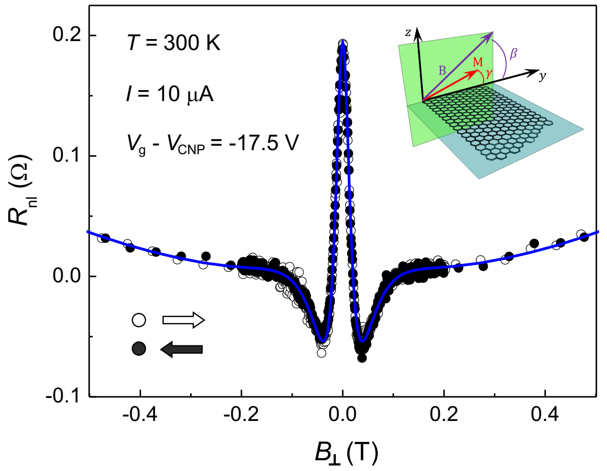

Supplementary Figure 4. Spin precession and determination of the tilting angle of the electrodes magnetization. Conventional spin precession measurements $R_{\rm nl}$ vs $B_\perp$ for down (solid circles) and up (open circles) field sweeps. The line is a least square fit using the Hanle expression for $\beta = 90°$, which is extended to include the tilting of the magnetization of the electrodes using Supplementary Equation 3. From the fit, the saturation field $B_{\rm s}$ is obtained[1,2]. The inset shows the orientations of the magnetization of the electrodes and the applied magnetic field in the yz plane.

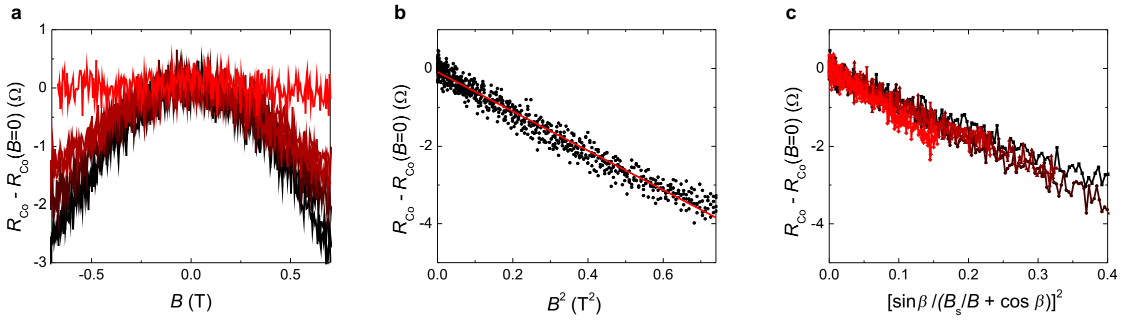

Supplementary Figure 5. Anisotropic magnetoresistance in a Co electrode. **a**, $R_{\mathrm{Co}} - R_{\mathrm{Co}}(B = 0)$ versus B for $\beta = 0°, 60°, 70°, 90°$ (top to bottom). **b**, $R_{\mathrm{Co}} - R_{\mathrm{Co}}(B = 0)$ versus B^2 at $\beta = 90°$ demonstrating the parabolic dependence predicted by Supplementary Equation 8. **c**, $R_{\mathrm{Co}} - R_{\mathrm{Co}}(B = 0)$ versus $\{\sin(\beta)/[B_{\mathrm{s}}B^{-1} + \cos(\beta)]\}^2$ at β, demonstrating the dependence predicted by Supplementary Equation 7.

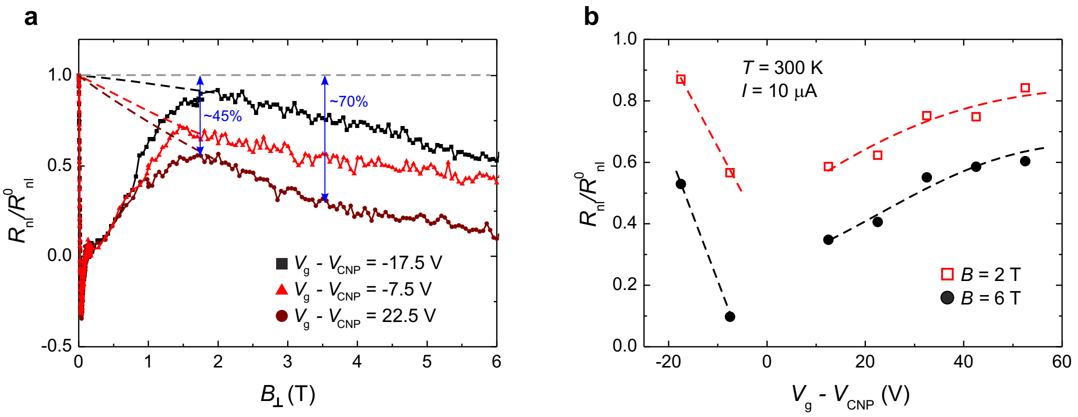

Supplementary Figure 6. Non-local resistance R_{nl} as a function of applied magnetic field $B_\perp$. **a**, R_{nl} shows typical spin precession (Hanle) measurements at low fields; R_{nl} appears to saturate at high fields (~ 1.5 T) when the magnetization of the electrodes point out-of-plane. Further increase of $B_\perp$ demonstrates that R_{nl} has not saturated as it decreases monotonously after reaching a maximum. The dashed lines show rough extrapolations of the nonlocal resistance from the magnetization saturation region down to $B = 0$, which demonstrate that isotropic spin relaxation cannot be excluded. **b**, R_{nl} at the indicated magnetic fields versus V_{g}. The non-local resistance depends significantly on the carrier density (tuned by V_{g}) and on the applied B for all V_{g}.

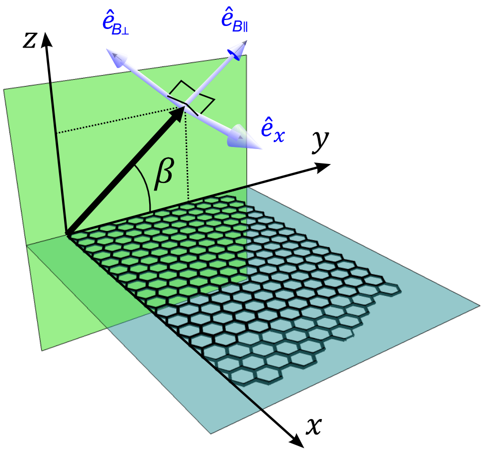

Supplementary Figure 7. Cartesian axis used for the calculation of the spatial evolution of the spin density. The unit vector $\hat{\mathbf{e}}_x$ is along the spin-propagation channel, while $\hat{\mathbf{e}}_{B_\parallel}$ and $\hat{\mathbf{e}}_{B_\perp}$ fix the parallel and perpendicular directions to the magnetic field $\mathbf{B}$, respectively.

II. SUPPLEMENTARY NOTES

Supplementary Note 1. Electrical characterization

Supplementary Figure 2a shows the square resistance $R_{\rm sq}$ of the graphene channel at $T =$ 300 K as a function of the gate voltage $V_{\rm g}$. Here $V_{\rm g}$ is applied to the p^{++}Si substrate, which acts as a backgate. We observe a pronounced electric-field effect from which we estimate an average electron/hole mobility $\mu = 1.7 \times 10^4$ cm^{2}V^{-1}s^{-1} and a residual carrier density $n_0 = 1.5 \times 10^{11}$cm^{-2}, demonstrating high sample quality[3]. The magnitude of n_0 defines the region where electron-hole puddles are present in graphene, resulting in an inhomogeneous carrier density that prevents a reliable extraction of transport parameters. Therefore, all of the extracted parameters reported in our work are well outside this region.

The spin transport characteristics of the device are presented in Fig. 2 of the main manuscript. Supplementary Figure 2b shows $\tau_{\rm s||}$ versus $D_{\rm s}$ with a linear relation between $D_{\rm s}$ and $\tau_{\rm s||}$. According to the common interpretation, this linear relationship would suggest that the EY mechanism of spin relaxation is dominant (with a spin-orbit strength of 0.6 meV, as typically found in the literature[4–6]). However, such phenomenological analysis is not sufficient to unambiguously identify the microscopic origin of the spin relaxation[7,8]. As discussed in the main text, changing the carrier density by means of an external gate can modify spin transport in an unintended way.

We note that nominally identical devices could differ from one laboratory to another due to variability in the concentration of adatoms, which might be present due to the implemented processing steps. For example, the contradictory results on spin dynamics in hydrogenated graphene can be rooted in the different procedures used to achieve the hydrogenation, or perhaps in intrinsic variations in the as-fabricated devices[5,9–11]. The same conclusion could be drawn regarding the experiments that study the spin transport properties after physical deposition of metals, such as gold, onto graphene[12–14]. It might be difficult to establish the effect of adding any specific adatom if the pre-existing spin relaxation mechanisms are not well known.

Supplementary Note 2. Graphene magnetoresistance

The application of a magnetic field can result in changes in the resistance of the graphene layer. The magnetoresistance is expected to become more important closer to the charge neutrality point (CNP) and in samples that have long mean free paths and large mobilities. In such samples, the orbital motion of the carriers could be strongly altered even when the applied magnetic field is of modest strength. It is therefore crucial to carefully study how the spin transport is modified by the magnetic field in order to eliminate possible measurement artifacts. In our model, the magnetoresistance effect is taken into account by the pre-factor $\alpha(B)$, which is a function of the square resistance R_{sq} and the effective polarization of the electrodes (see Methods).

Supplementary Figure 3 shows R_{sq} of graphene for different V_{g} as a function of the applied magnetic field perpendicular to the plane, $B_{\perp}$, up to 4 T. As expected, the magnetoresistance is more pronounced close to the CNP, $V_{\mathrm{g}} \sim V_{\mathrm{CNP}}$. There, variations can be beyond 35% at $B_{\perp} \sim 1$ T, in agreement with recent reports[15,16]. When the magnetic field drops below ~ 0.2 T, the variations are within 3%, independently of V_{g} (inset Supplementary Figure 3). Only then we find that the magnetic field does not introduce a significant change in our measurements. We thus require that all of the measurements are performed below 0.2 T, which then implies that the dephasing magnetic field B_{d} must be about 0.1 T or lower. This issue is further discussed in the Supplementary Note 6 in connection with the determination of $\tau_{\mathrm{s}\perp}$ with the technique introduced in refs. 15 and 17.

Supplementary Note 3. Device design: dephasing of the spin component perpendicular to the field and contact induced spin relaxation

The distance L between injector and detector has to be large enough so that complete dephasing of the component perpendicular to the magnetic field is induced at a low enough magnetic field strength B_{d}. As a rule of thumb[1,18], for complete dephasing, the spread of precession angles $\Delta\phi$ for the characteristic diffusion time $\tau_{\mathrm{diff}}(L)$ from the injector to the detector has to exceed 2π . When $\tau_{\mathrm{diff}}(L) \sim \tau_{\mathrm{s}}$ (*i.e.* $L \sim \sqrt{2}\lambda_{\mathrm{s}}$), this threshold condition can be written as $\Delta\phi = \omega_{\mathrm{L}}(B_{\mathrm{d}})\tau_{\mathrm{s}} = 2\pi$, where $\omega_{\mathrm{L}}(B_{\mathrm{d}})$ is the Larmor precession frequency at B_{d}. For typical graphene spin lifetimes $\tau_{\mathrm{s}} \sim 0.5$ ns, we obtain $B_{\mathrm{d}} \sim 70$ mT, which lies within the low B range necessary to keep marginal magnetoresistance effects (see Supplementary Note 2). If in our devices we choose $L \geq \sqrt{2}\lambda_{\mathrm{s}}$, $\tau_{\mathrm{diff}}(L)$ would exceed τ_{s}, therefore this criterion

indicates that $B_\mathrm{d} \sim 70$ mT is an estimation of the upper bound of the dephasing field.

In addition, the device design must be optimized to suppress the influence of the electrodes on the spin precession measurements and reduce the number of unknown parameters. Spin sinking at the contacts[19] can affect the determination of the spin relaxation time and has been a topic of recent debate (see, for example, refs. 20–24). In this works, it has been demonstrated (both theoretically and experimentally) that the influence of the electrodes can be minimized by having *i)* large contact resistances R_c and *ii)* a separation between injector and detector L that is substantially larger than λ. The argument is simple: the contact resistance reduces the flow of spins between the metallic electrodes and graphene, whereas for large L most of the spin relaxation and diffusion happens in the graphene without interference of the electrodes. As described in the preceding paragraph, a large L is already a critical requirement for the spin anisotropy measurements. Large contact resistances must also be introduced to achieve an efficient spin injection. Indeed, using available models and the device characteristics, $L = 11$ μm, $R_\mathrm{c} \gtrsim 10$ kΩ, $\lambda = 2 - 5.8$ μm (Fig. 3c, main text), and R_sq (Supplementary Figure 2a), we estimate[20–24] that the presence of the contacts will reduce the spin lifetime in no more than 10%. Such estimations are supported by recent experimental results using samples of similar characteristics, where no influence of the contacts was observed[15,25].

Considering an effective contact-induced spin relaxation rate Γ_c, then the measured in-plane and out-of-plane spin lifetimes would be given by $(\Gamma_\parallel + \Gamma_\mathrm{c})^{-1}$ and $(\Gamma_\perp + \Gamma_\mathrm{c})^{-1}$, respectively, where $\Gamma_\parallel$ and $\Gamma_\perp$ are the graphene in-plane and out-of-plane spin relaxation rates and, as discussed above, $\Gamma_\mathrm{c}/\Gamma_\parallel \lesssim 0.1$. When considering the influence of the contacts, the fitted anisotropy ratio ζ is then,

$$\zeta = \frac{\Gamma_\parallel + \Gamma_\mathrm{c}}{\Gamma_\perp + \Gamma_\mathrm{c}} = \zeta^\mathrm{G} \frac{1+\kappa}{1+\zeta^\mathrm{G}\kappa} \approx \zeta^\mathrm{G}[1 + \kappa(1-\zeta^\mathrm{G})], \quad (1)$$

where $\zeta^\mathrm{G} = \Gamma_\parallel/\Gamma_\perp$ is the actual anisotropy ratio of graphene, and $\kappa = \Gamma_\mathrm{c}/\Gamma_\parallel$.

Using the above expression we observe that ζ is rather insensitive to the presence of moderate Γ_c. For the upper limit of $\kappa = 0.1$ when $\zeta^\mathrm{G} = 0.9$, we obtain $\zeta \approx 0.908$, which underestimates ζ^G by less than 1%. For larger anisotropies, ζ further deviates from ζ^G.

However, even when $\zeta^{\mathrm{G}} = 0.5$, $\zeta \approx 0.52$ and the difference between them is still below 5%.

Supplementary Note 4. Magnetization orientation of the injector and detector electrodes from spin precession measurements

In order to determine the anisotropy accurately, it is necessary to have into account the tilting of the magnetization of the Co electrodes when subjected to the applied magnetic field B. To describe the magnetization rotation, we use the Stoner-Wohlfarth model, which provides a very good approximation for coherent magnetization rotation at low B, as demonstrated below. Within this model, the total energy density of the system, U, is given by the sum of the magnetostatic and anisotropy energies[26]

$$U = \kappa_{\mathrm{eff}} \sin^2(\gamma) - \mu_0 H M_{\mathrm{s}} \cos(\beta - \gamma), \tag{2}$$

where M_{s} is the saturation magnetization and κ_{eff} is an effective anisotropy constant; β is the angle between the external magnetic field and the easy (long) axis of the ferromagnet, while γ denotes the angle between the magnetization direction and the easy axis (see inset of Supplementary Figure 4). For a given β, the value of γ is obtained by minimizing U in Supplementary Equation 2.

For the specific case, $\beta = 90°$, the minimization results in,

$$\gamma = \arcsin(B/B_{\mathrm{s}}), \tag{3}$$

with $\mu_0 H = B$ and $B_{\mathrm{s}} = 2\kappa_{\mathrm{eff}}/M_{\mathrm{s}}$, the saturation field. This relationship is known to closely follow experimental results[2,27], even for magnetic fields approaching B_{s}. When considering the tilting in the conventional spin precession measurements ($\beta = 90°$), the agreement with the experimental data is excellent (see Supplementary Figure 4); from the fitting we obtain $B_{\mathrm{s}} = 1.12$ T and a small magnetization rotation out of the graphene plane of $\gamma < 5°$ for $B < B_{\mathrm{d}}$.

For arbitrary β, the minimization of U leads to,

$$\gamma = \arcsin\left[\frac{\sin(\beta)}{\frac{B_{\mathrm{s}}}{B} + \cos(\beta)}\right], \tag{4}$$

where we assumed that γ is small, and thus $\cos(\gamma) \approx 1$. Here, $B_{\mathrm{s}} = 1.12$ T, as obtained from the $\beta = 90°$ case. Note that for small $B \ll B_{\mathrm{s}}$, Supplementary Equation 4 reduces to Supplementary Equation 3, with the component of the magnetic field perpendicular to the substrate, $B\sin(\beta)$, replacing B.

Supplementary Note 5. Magnetization orientation of the injector and detector electrodes from anisotropic magnetoresistance measurements

Besides the spin precession measurements, we have additionally checked the functional dependence of γ on B and β by performing anisotropic magnetoresistance (AMR) measurements on a Co bar. The resistivity ρ of a Co film is larger by a few percent when the magnetization and the current directions are parallel than when they are perpendicular, that is, $\rho_{\|} > \rho_{\perp}$. For an arbitrary angle ψ between the current and the magnetization directions, ρ follows,

$$\rho(\psi) = \rho_{\perp} + \Delta\rho\cos^2(\psi), \tag{5}$$

where $\Delta\rho = \rho_{\|} - \rho_{\perp}$.

At $B = 0$, the magnetization of Co is along its long axis and the applied current is parallel to it ($\psi = 0$), therefore, $\rho(B = 0) = \rho_{\perp} + \Delta\rho$. When $B \neq 0$ is applied at an angle β, the magnetization tilts by γ, therefore, the angle ψ between the current and the magnetization is $\psi = \gamma(\beta, B)$. By noting that $\sin^2(\gamma) + \cos^2(\gamma) = 1$ and using $\rho(B = 0) = \rho_{\perp} + \Delta\rho$, Supplementary Equation 5 can be rewritten as,

$$\rho(\beta, B) - \rho(B = 0) = -\Delta\rho\sin^2[\gamma(\beta, B)] \tag{6}$$

By replacing $\sin^2[\gamma(\beta, B)]$ in Supplementary Equation 6 using Supplementary Equation 4, we find,

$$\rho(\beta, B) - \rho(B=0) = -\Delta\rho\Big[\frac{\sin(\beta)}{\frac{B_{\rm s}}{B} + \cos(\beta)}\Big]^2. \quad (7)$$

For $\beta = 90°$, the above expression reduces to a simple quadratic relationship,

$$\rho(\beta, B) - \rho(B=0) = -\frac{\Delta\rho}{B_{\rm s}^2}B^2 \quad (8)$$

Supplementary Figure 5a shows AMR measurements $R_{\rm Co} - R_{\rm Co}(B=0)$ vs B for various values of β. Supplementary Figure 5b presents the measurements for $\beta = 90°$ vs B^2 showing that the quadratic relationship Supplementary Equation 8 holds up to at least 800 mT. Finally, Supplementary Figure 5c presents the data in Supplementary Figure 5a plotted versus $\{\sin(\beta)/[B_{\rm s}B^{-1} + \cos(\beta)]\}^2$. As expected from Supplementary Equation 7, it is clearly observed that all of the data fall in a universal line with negative slope. This further confirms that $\gamma(\beta, B)$ can be determined quantitatively using Supplementary Equation 4.

Supplementary Note 6. Determination of $\tau_{\rm s\perp}$ with perpendicularly magnetized electrodes

In refs. 15 and 17, the anisotropy is calculated by performing non-local measurements with $\beta = 90°$. First $\tau_{\rm s\|}$ is determined by conventional spin precession measurements as in Fig. 1a (main manuscript). Subsequently, $\tau_{\rm s\perp}$ is determined by increasing $B_\perp$ to above 1 T, forcing the magnetization direction of the electrodes to align to the field and, therefore, enabling the injection of spins perpendicular to the graphene plane. In this case Equation 7 (Methods) reduces to,

$$\frac{R_{\rm nl}(B)}{R_{\rm nl}(B=0)} = \frac{\alpha(B)}{\alpha(B=0)}\sqrt{\zeta}e^{-\sqrt{\frac{L^2}{\tau_{\rm s\|}D_{\rm s}}}(\sqrt{\frac{1}{\zeta}}-1)}. \quad (9)$$

Because $\tau_{\rm s\|}$ can be determined by fitting the spin precession response at low magnetic fields,

Supplementary Equation 9 provides a method to extract the spin relaxation anisotropy if α is known. However, because the magnetoresistance in single-layer graphene can be very large, one cannot assume that the square resistance, R_{sq}, and associated diffusion constants are field independent over the whole carrier density range[15,16,28]. This constrains the range of applicability of Supplementary Equation 9 for the determination of the spin relaxation anisotropy, as pointed out in ref. 15.

Supplementary Figure 6a shows our measurements of R_{nl} vs $B_{\perp}$. We use the device of the main text in order to compare our anisotropy results with those obtained with the method in refs. 15 and 17. We observe that R_{nl} appears to start saturating at a large magnetic field, $B_{\perp} \sim 1$ T, indicating complete rotation of the magnetization of the electrodes[2,15,17]. However, further increase of $B_{\perp}$ demonstrates that R_{nl} does not really saturate, presenting a monotonous decrease after reaching a maximum value at $B_{\perp} \sim 1.5$ T; the exact position of the maximum depends on V_{g}, and the decrease does not slow down as the magnetic field increases. This behaviour is observed even at large $n = 2.7 \times 10^{12}$ cm^{-2} and suggests that the pre-factor α in Supplementary Equation 9 and/or the diffusion constant might be field-dependent even at lower magnetic fields.

For our sample, it is therefore not possible to directly neglect magnetoresistive effects to determine the spin-lifetime anisotropy with Supplementary Equation 9. For instance, due to the magnetoresistance, if we use Supplementary Equation 9 the calculated value of ζ at $V_{\mathrm{g}} - V_{\mathrm{CNP}} = 22.5$ V would be 0.67 at $B_{\perp} \sim 1.75$ T and 0.55 at $B_{\perp} \sim 3.5$ T. By extrapolating the field dependence observed in Supplementary Figure 6a to $B = 0$, it becomes apparent that the anisotropy would in fact be overestimated if Supplementary Equation 9 was used. Moreover, an anisotropy ratio $\zeta = 1$ is not incompatible with these measurements, as schematically shown with the dashed lines in Supplementary Figure 6a.

Knowing the graphene magnetoresistance, we propose a quick check to determine the suitability of the perpendicular spin-injection method to determine ζ. The magnetoresistance shows a parabolic dependence for low magnetic field magnitude ($\lesssim 0.5$ T), but departs from this dependence at sufficiently large magnetic fields, most notably close to the CNP. The dependence becomes approximately linear, or even sub-linear at the CNP but, in general, the change in R_{sq} between $B_{\perp} = 0$ and $B_{\perp} = B_{\perp}^{\mathrm{sat}}$ (with $B_{\perp}^{\mathrm{sat}} > 1$ T) is of the same order of magnitude that the change between $B_{\perp} = B_{\perp}^{\mathrm{sat}}$ and $B_{\perp} = 2 \times B_{\perp}^{\mathrm{sat}}$, for all values of V_{g}. Therefore, assuming that the effect of the magnetoresistance is small, and considering

first order changes in R_{nl} as a consequence of it, then the change in R_{nl} between $B_{\perp} = 0$ and $B_{\perp} = B_{\perp}^{\mathrm{sat}}$ should be of the same order of magnitude as the change between $B_{\perp} = B_{\perp}^{\mathrm{sat}}$ and $B_{\perp} = 2 \times B_{\perp}^{\mathrm{sat}}$ (note that we are ignoring quantum transport effects that could be significant in high mobility samples at $B \sim 1$ T or even lower).

With this in mind, a necessary condition that has to be fulfilled for the perpendicular spin-injection method to be reliable is that the measurements of $\tau_{\mathrm{s}\perp}$ at the saturation magnetic field, $B_{\perp}^{\mathrm{sat}}$, and at $2 \times B_{\perp}^{\mathrm{sat}}$ must yield the same results, which is equivalent to saying that R_{nl} stays constant between $B_{\perp}^{\mathrm{sat}}$ and $2 \times B_{\perp}^{\mathrm{sat}}$. This criterion is obviously not met by our data (or the data in ref. 17) despite the fact that the overall change in R_{sq} is below 20% at $B_{\perp}^{\mathrm{sat}}$ (compare Supplementary Figures 3 (inset) and 6). The data presented in ref. 15 does not extend to large enough magnetic fields to test this criterion.

The same criterion can be applied to our technique. The situation is significantly more favorable in this case because of the parabolic dependence of R_{sq} vs $B_{\perp}$, which results in a magnetoresistance that is below 2% at 0.2 T, except within a narrow range of n about the CNP (note that even at the CNP, the effect is below 3%, see Supplementary Figure 3 inset). As shown in Figs. 3b and 4a of the main text, R_{nl} stays nearly constant over a broad magnetic field range. When R_{nl} is carefully analyzed, a small increase with magnetic field is observed. However, this change can be quantitatively accounted for by the small tilting γ of the ferromagnetic electrodes induced by B, as discussed in Supplementary Notes 4 and 5.

III. SUPPLEMENTARY REFERENCES

[1] Jedema, F. J., Heersche, H. B., Filip, A. T., Baselmans, J. J. A. & van Wees, B. J. Electrical detection of spin precession in a metallic mesoscopic spin valve. *Nature* **416,** 713-716 (2002).

[2] Valenzuela, S. O. & Tinkham, M. Direct electronic measurement of the spin Hall effect. *Nature* **442,** 176-179 (2006).

[3] Zhong, H., Zhang, Z., Xu, H., Qiu, C. & Peng, L.-M. Comparison of mobility extraction methods based on field-effect measurements for graphene. *AIP Advances* **5,** 057136 (2015).

[4] Zomer, P. J., Guimarães, M. H. D., Tombros, N. & van Wees, B. J. Long-distance spin transport in high-mobility graphene on hexagonal boron nitride. *Phys. Rev. B* **86,** 161416(R) (2012).

[5] Balakrishnan, J., Koon, G. k. W., Jaiswal, M., Castro Neto, A. H. & Özyilmaz, B. J. Colossal enhancement of spin-orbit coupling in weakly hydrogenated graphene. *Nat. Phys.* **9,** 284-287 (2013).

[6] Kamalakar, M.V., Groenveld, C., Dankert, A. & Dash, S.P. Long distance spin communication in chemical vapour deposited graphene. *Nat. Commun.* **6,** 6766 (2015).

[7] Han, W., Kawakami,R. K., Gmitra, M. & Fabian, J. Graphene spintronics. *Nat. Nanotechnol.* **9,** 324-340 (2014).

[8] Roche, S. & Valenzuela, S. O. Graphene spintronics: puzzling controversies and challenges for spin manipulation. *J. Phys. D* **47,** 094011 (2014).

[9] McCreary, K. M., Swartz, A. G., Han, W., Fabian, J. & Kawakami, R. K. Magnetic moment formation in graphene detected by dcattering of pure spin currents. *Phys. Rev. Lett.* **109,** 186604 (2012).

[10] Wojtaszek, M., Vera-Marun, I. J., Maassen, T. & van Wees, B. J. Enhancement of spin relaxation time in hydrogenated graphene spin-valve devices. *Phys. Rev. B* **87,** 081402(R) (2013).

[11] Kaverzin, A. A. & van Wees, B. J. Electron transport nonlocality in monolayer graphene modified with hydrogen silsesquioxane polymerization. *Phys. Rev. B* **91,** 165412 (2015).

[12] Pi, K., Han, W., McCreary, K. M., Swartz, A. G., Li, Y. & Kawakami, R. K. Manipulation of spin transport in graphene by surface chemical doping. *Phys. Rev. Lett.* **104,** 187201 (2010).

[13] Balakrishnan, J., Koon, G. k. W., Avsar, A., Ho, J., Lee, H. J., Jaiswal, M., Baeck, S.- J., Ahn, J.- H., Ferreira, A., Castro Neto, A. H. & Özyilmaz, B. Giant spin Hall effect in graphene grown by chemical vapour deposition. *Nat. Commun.* **5,** 4748 (2014).

[14] Wang, Y., Cai, X., Reutt-Robey, J. & Fuhrer, M. S. Neutral-current Hall effects in disordered graphene. *Phys. Rev. B* **92,** 161411(R) (2015).

[15] Guimarães, M. H. D., Zomer, P. J., Ingla-Aynés, J., Brant, J. C., Tombros, N. & van Wees, B. J. Controlling spin relaxation in hexagonal BN-encapsulated graphene with a transverse electric field. *Phys. Rev. Lett.* **113,** 086602 (2014).

[16] Cho, S. & Fuhrer, M. S. Charge transport and inhomogeneity near the minimum conductivity point in graphene. *Phys. Rev. B* **77,** 081402R (2008).

[17] Tombros, N., Tanabe, S., Veligura, A., Jozsa, C., Popinciuc, M., Jonkman, H. T. & van Wees, B. J. Anisotropic spin relaxation in graphene. *Phys. Rev. Lett.* **101,** 046601 (2008).

[18] Johnson, M. & Silsbee, R. H. Coupling of electronic charge and spin at a ferromagnetic-

paramagnetic metal interface. *Phys. Rev. Lett.* **37,** 5312-5325 (1988).

[19] Takahashi, S. & Maekawa, S. Spin injection and detection in magnetic nanostructures. *Phys. Rev. B* **67,** 052409 (2003).

[20] Maassen, T., Vera-Marun, I. J., Guimarães, M. H. D. & van Wees, B. J. Contact-induced spin relaxation in Hanle spin precession measurements. *Phys. Rev. B* **86,** 235408 (2012).

[21] Sosenko, E., Wei, H.& Aji, V. Effect of contacts on spin lifetime measurements in graphene. *Phys. Rev. B* **89,** 245436 (2014).

[22] Volmer, F., Drögeler, M., Maynicke, E., von den Driesch, N., Boschen, M. L., Güntherodt, G., Stampfer, C. & Beschoten, B. Suppression of contact-induced spin dephasing in graphene/MgO/Co spin-valve devices by successive oxygen treatments. *Phys. Rev. B* **90,** 165403 (2014).

[23] Idzuchi, H., Fert, A. & Otani, Y. Revisiting the measurement of the spin relaxation time in graphene-based devices. *Phys. Rev. B* **91,** 241407(R) (2015).

[24] Amamou, W., Lin, Z., van Baren, J., Turkyilmaz, S., Shi, J. & Kawakami, R. K. Contact induced spin relaxation in graphene spin valves with Al_2O_3 and MgO tunnel barriers. Preprint at http://arxiv.org/abs/1512.02255 (2015).

[25] Guimarães, M. H. D., Veligura, A., Zomer, P. J., Maassen, T., Vera-Marun, I. J., Tombros, N. & van Wees, B. J. Spin transport in high-quality suspended graphene devices. *Nano Lett.* **12,** 3512-3517 (2012).

[26] Blundell, S. Magnetism in Condensed Matter. (Oxford Master Series in Physics) (Oxford University Press, USA, 2001).

[27] Motsnyi, V. F., Van Dorpe, P., Van Roy, W., Goovaerts, E., Safarov, V. I., Borghs, G. & De Boeck, J. Optical investigation of electrical spin injection into semiconductors. *Phys. Rev. B* **68,** 245319 (2003).

[28] Tomaru, T., Fujii, K., Ohyama, T. & Otsuka, E. Magnetic-field effect on the diffusion of nonequilibrium carriers in germanium. *Phys. Rev. B* **42,** 9104-9112 (1990).
